# Leukotriene Involvement in the Insulin Receptor Pathway and Macrophage Profiles in Muscles from Type 1 Diabetic Mice

**DOI:** 10.1155/2019/4596127

**Published:** 2019-01-27

**Authors:** João Pedro Tôrres Guimarães, Luciano Ribeiro Filgueiras, Joilson Oliveira Martins, Sonia Jancar

**Affiliations:** ^1^Laboratory of Immunopharmacology, Department of Immunology, Institute of Biomedical Sciences, University of São Paulo (ICB/USP), São Paulo, Brazil; ^2^Laboratory of Immunoendocrinology, Department of Clinical and Toxicological Analyses, School of Pharmaceutical Sciences of University São Paulo (FCF/USP), São Paulo, Brazil

## Abstract

Type 1 diabetes (T1D) is a metabolic disease associated with systemic low-grade inflammation and macrophage reprogramming. There is evidence that this inflammation depends on the increased systemic levels of leukotriene (LT) B4 found in T1D mice, which shifts macrophages towards the proinflammatory (M1) phenotype. Although T1D can be corrected by insulin administration, over time T1D patients can develop insulin resistance that hinders glycemic control. Here, we sought to investigate the role of leukotrienes (LTs) in a metabolically active tissue such as muscle, focusing on the insulin signaling pathway and muscle-associated macrophage profiles. Type 1 diabetes was induced in the 129/SvE mouse strain by streptozotocin (STZ) in mice deficient in the enzyme responsible for LT synthesis (5LO^−/−^) and the LT-sufficient wild type (WT). The response to insulin was evaluated by the insulin tolerance test (ITT), insulin concentration by ELISA, and Akt phosphorylation by western blotting. The gene expression levels of the insulin receptor and macrophage markers Stat1, MCP-1, Ym1, Arg1, and IL-6 were evaluated by qPCR, and that of IL-10 by ELISA. We observed that after administration of a single dose of insulin to diabetic mice, the reduction in glycemia was more pronounced in 5LO^−/−^ than in WT mice. When muscle homogenates were analyzed, diabetic 5LO^−/−^ mice showed a higher expression of the insulin receptor gene and higher Akt phosphorylation. Moreover, in muscle homogenates from diabetic 5LO^−/−^ mice, the expression of anti-inflammatory macrophage markers *Ym1*, *Arg1*, and IL-10 was increased, and the relative expression of the proinflammatory cytokine *IL-6* was reduced compared with WT diabetic mice. These results suggest that LTs have an impact on the insulin receptor signaling pathway and modulate the inflammatory profile of muscle-resident macrophages from T1D mice.

## 1. Introduction

The incidence of metabolic disorders is increasing dramatically and is now widely considered a serious threat to public health. In diseases such as diabetes, obesity, atherosclerosis, and gout, metabolic imbalance is associated with the establishment of low-grade systemic inflammation, which in turn is a determining factor in the pathophysiology of these diseases. This happens as a consequence of the accumulation of certain metabolic products, such as glucose, fatty acids, uric acid, and cholesterol, which activate receptors of innate immunity in leukocytes and induce the chronic production of proinflammatory cytokines and lipid mediators [1–3].

Characterized by chronic hyperglycemia with changes in the metabolism of carbohydrates, lipids, and proteins [4], diabetes is classically divided into two forms. In type 2 diabetes (T2D), hyperglycemia is due to insulin resistance established in the liver, muscle, and adipose tissue, and the main risk factor for this condition is obesity [5]. In T1D, hyperglycemia results from deficient insulin production as a consequence of the destruction of pancreatic *β* cells by autoimmune processes. This condition is corrected by insulin administration, but throughout treatment, T1D patients also begin to develop resistance to insulin, and glycemic control becomes increasingly difficult, which impairs the patient's quality of life [6]. It is believed that in both T1D and T2D, insulin resistance is due to a systemic low-grade inflammation; however, the mechanisms involved may be distinct and still need to be elucidated.

In muscles, the accumulation of lipids along with their peroxidation promotes endoplasmic reticulum stress, and muscle-associated macrophages undergo reprogramming to the proinflammatory profile, producing IL-6, IL-1*β*, TNF-*α*, and lipid mediators [7–10]. The high level of TNF-*α* produced by these macrophages stimulates production of the chemokine CCL2, leading to the recruitment of activated monocytes (CD11b + LY6C^high^) to the tissue [11]. By binding to their membrane receptors on muscle cells, the cytokines IL-6, IL-1*β*, and TNF-*α* can induce insulin resistance [10, 12].

The lipid mediator leukotriene B4 (LTB4) plays a central role in systemic low-grade inflammation [13–15] and the establishment of insulin resistance in animal models of diabetes [10, 16, 17]. Leukotrienes (LTs) are generated from arachidonic acid (AA) metabolism by 5-lipoxygenase (5LO). Arachidonic acid is esterified in cell membrane phospholipids from where it is released by activated phospholipase PLA2. Together with other enzymes of the 5LO metabolic pathway, macrophages and other inflammatory cells are able to generate high amounts of LTs within a few minutes of stimulation. Together with the accessory protein FLAP (5-lipoxygenase-activating protein), 5LO oxidizes AA, generating the unstable intermediate LTA4, which is rapidly hydrolyzed to generate LTB4 [16]. LTB4 binds to G protein-coupled receptors; BLT1 is the high-affinity receptor and is coupled to Gi protein, thereby resulting in decreased intracellular levels of cyclic AMP.

Activation of the BLT1 receptor in macrophages potentiates phagocytosis, microbicidal activity, and the production of proinflammatory cytokines [2]. This proinflammatory profile of macrophages in T1D mice is associated with increased levels of LTB4 in the blood, systemic inflammation, and insulin resistance. Blocking of LTs shifts macrophages towards an anti-inflammatory profile and reduces systemic inflammation and insulin resistance. When sepsis was induced in these animals, the systemic inflammatory response was more intense, and animal mortality was increased. This was reversed by LT antagonists [17].

Recently, it has been demonstrated that in mice fed a high-fat diet, LTB4 is produced in adipose tissue, muscles, and liver. Adipose tissue macrophages exhibit a proinflammatory profile and insulin resistance. Similarly, in the liver and muscle of obese mice, LTB4 promotes inflammation and insulin resistance [10, 16–20]. Therefore, considering studies involving LTs in inflammation and how they may be involved in the development of metabolic syndromes, in the present study we investigated the participation of LTs in the insulin receptor pathway, an important checkpoint pathway related with insulin resistance, and the macrophage profile, an important cell in inflammatory processes.

## 2. Materials and Methods

### 2.1. Animals

We used 8-week-old, male, pathogen-free 129/SvE wild-type (WT) and 5LO knockout (5LO^−/−^) mice. The animals were maintained in a controlled environment at 22°C under a 12-hour light-dark cycle with free access to water and restricted access to food only before the T1D induction protocol, as described below. This study was carried out in strict accordance with the principles and guidelines adopted by the Brazilian National Council for the Control of Animal Experimentation (CONCEA) and approved by the Ethical Committee on Animal Use (CEUA) of the Institute of Biomedical Sciences (ICB) of the University of São Paulo (USP) (CEUA no. 08/2014). All surgical procedures were performed under ketamine/xylazine hydrochloride anesthesia, and care was taken to minimize animal suffering.

### 2.2. Induction of Diabetes Mellitus

For the induction of T1D, the animals were fasted for 5 hours, followed by an intraperitoneal (i.p.) injection of STZ (ChemCruz® U-9889, lot F1816) (30 mg/kg for WT and 25 mg/kg for KO, in 0.1 M citrate buffer, pH 4.5); after that, the animals were maintained with free access to food. This protocol lasted for a period of 5 consecutive days. Animals with glycemia greater than 300 mg/dL (OneTouch® Select Simple™) 10 days after the last dose were considered diabetic. Mice in the nondiabetic control group received a citrate buffer injection alone. To evaluate variation in body weight, the animals were weighed before and 10 and 17 days after STZ administration. The STZ-induced destruction of pancreatic beta cells is commonly accepted as a model of T1D, has been described by other groups [21, 22], and has also been previously standardized in our laboratory [17, 23].

### 2.3. Insulin Tolerance Test (ITT)

After a 6-hour fast, the mice received an i.p. injection of insulin (Novolin®, lot CS6G140, 1.0 IU/kg), and the blood glucose level was determined (OneTouch® Select Simple™) using blood samples collected from the caudal vein every 30 minutes.

### 2.4. Insulin-Induced AKT Phosphorylation

The animals were anaesthetized with ketamine/xylazine (90 mg/kg and 10 mg/kg, respectively) and 5 minutes after the i.p. insulin injection, tissue samples were collected and macerated (Polytron PT 1600E) in RIPA buffer (50 mM Tris, pH 8.0, 150 mM NaCl, 1% Triton X-100, and 0.1% SDS) containing a protease inhibitor (Sigma-Aldrich), orthovanadate, and fluoride and subsequently centrifuged (1500 × g for 5 minutes).

### 2.5. Measurement of Serum Insulin Levels

After the collection of whole blood from T1D and control mice, the samples were centrifuged for 20 minutes at 1600 × g (for serum separation). The serum insulin concentration was determined using an insulin kit (Insulin Mouse ELISA Kit, lot 534070518, Thermo Fisher Scientific). The test is based on the capacity of the insulin in the sample to compete with the acetylcholinesterase-conjugated insulin for specific antibody binding. The reading was performed at 414 nm on a microplate reader (Epoch Microplate Spectrophotometer, BioTek Instruments), and the results are expressed in ng/mL.

### 2.6. Western Blotting

Protein concentration in the samples was determined (BCA Assay Kit, Thermo Fisher Scientific). Equal amounts of protein were separated by electrophoresis (SDS-PAGE) and transferred to a nitrocellulose membrane. After blocking of nonspecific binding (nonfat dried skim milk powder), the membranes were incubated overnight at 4°C under constant stirring with primary antibodies specific for pAkt Ser 473 (1 : 2000, Cell Signaling Technology, lot 0019) and *β* actin (1 : 1000, Cell Signaling Technology, lot 0017). Anti-rabbit IgG (1 : 3000, Cell Signaling Technology, lot 0025) was used as a secondary antibody. Expression was visualized using SuperSignal® West Pico Chemiluminescent Substrate (lot PD202858, Thermo Fisher Scientific).

### 2.7. RNA Purification and Real-Time PCR Analysis

Total RNA from the tissue homogenates was extracted as previously described [24]. cDNA was synthesized using the RevertAid First Strand cDNA Synthesis Kit (lot 00376305, Thermo Fisher Scientific), and qPCR was performed using primers for *Irs1*, *Insr*, *Il-6*, *Arg1*, *Stat1*, *Ym1*, and *Mcp1* (all from Exxtend®) on the Applied Biosystems StepOnePlus™ Real-Time PCR System (Thermo Fisher Scientific). Relative expression was calculated using the comparative threshold cycle (Ct) and expressed relative to the controls (ΔΔCt method).

### 2.8. Muscle Homogenates

Tissue samples were separately collected from the mice and homogenized in RIPA buffer with a tissue homogenizer (Polytron PT 1600E). The supernatants were separated from the cellular debris by centrifugation at 500 × g for 10 minutes, collected, and stored at −80°C. The protein concentration in the homogenates was determined using a commercial kit (Pierce™ BCA Protein Assay Kit, lot PE203766, Thermo Fisher Scientific). The assay was performed according to the manufacturer's manual. Absorbance values at 562 nm were obtained using a microplate reader (SpectraMax 190 Microplate Reader).

### 2.9. Cytokine Quantifications

The concentrations of IL-10 in the supernatants of tissue homogenates were measured using a BD OptEIA™ ELISA Set (BD Biosciences) following the manufacturer's protocol.

### 2.10. Data Analysis

Data were processed and analyzed by analysis of variance (ANOVA) and the Bonferroni posttest or unpaired *t*-tests using GraphPad Prism 6.0 software (La Jolla, CA, USA). Two-tailed *p* values with 95% confidence intervals were acquired. Data are represented as the mean ± standard error of the mean (SEM). Values of *p* < 0.05 were considered significant.

## 3. Results

### 3.1. Characterization of the T1D Model

**Figure 1:**
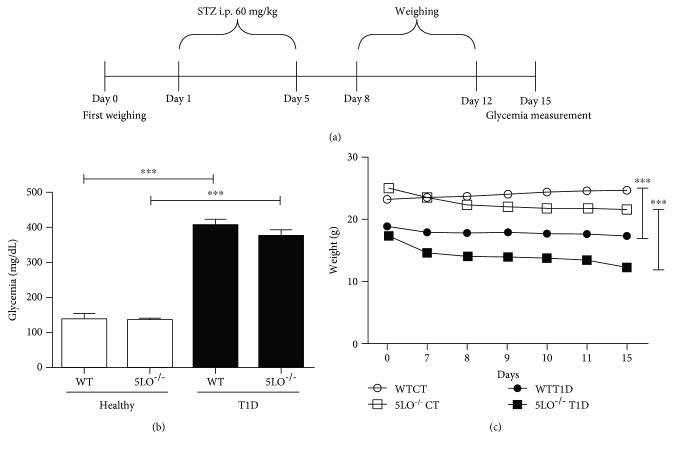
Characterization of T1D. (a) T1D induction protocol: 5LO^−/−^ and WT mice were fasted for 5 hours before daily injections (i.p.) of streptozotocin (60 mg/kg) for 5 days. Ten days after the last STZ injection, mouse blood glucose levels were measured, and mice with levels higher than 300 mg/dL were considered diabetic. The nondiabetic (healthy) group received the drug diluent, citrate buffer. (b) Blood glucose levels in T1D and healthy groups at day 15. (c) Mice were weighed before streptozotocin administration and 14 days after the last dose. *n* = 4-7 animals in each group and values are the mean ± SEM. ^∗∗∗^*p* < 0.001.

Figure 1 illustrates the experimental protocol employed. In WT and 5LO^−/−^ mice, STZ (60 mg/kg) was administered by i.p. injection in 5 doses over 5 days. Before each dose, the mice were fasted for 5 hours, and for 30 minutes after the injection, they were maintained with free access to food and water (Figure 1(a)). Blood glucose levels were measured three days after the last dose of STZ, and only mice with glycemia greater than 300 mg/dL were used in the experiments (Figure 1(b)). Body weight loss is one of the symptoms of T1D, and in our study both WT and 5LO^−/−^ diabetic mice showed significant body weight loss compared with healthy mice (Figure 1(c)).

### 3.2. Insulin Signaling Pathway in T1D

**Figure 2:**
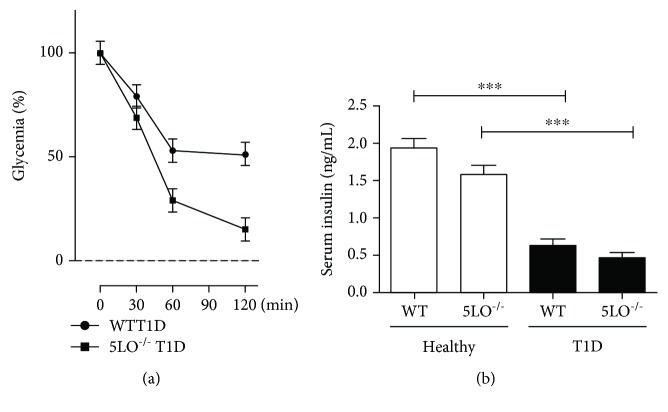
Insulin pathway in T1D serum. After insulin (1.0 IU/kg) administration, serum was taken from mice to analyze serum glucose (a) and insulin levels (b). *n* = 4-5 animals in each group and values are the mean ± SEM. ^∗^*p* < 0.05; ^∗∗∗^*p* < 0.001.

Diabetic mice were submitted to the insulin tolerance test. After i.p. injection of insulin (1.0 IU/kg), the blood glucose level was determined every 30 minutes. It was observed that diabetic WT mice presented difficulty controlling glucose levels over time, whereas the 5LO^−/−^ diabetic mice were able to control their blood glucose, suggesting a role for LTs in the insulin pathway (Figure 2(a)). When the production of insulin was evaluated in the serum, it was observed that both groups of diabetic mice (5LO^−/−^ and WT) presented significantly lower insulin content when compared with the nondiabetic controls (Figure 2(b)).

**Figure 3:**
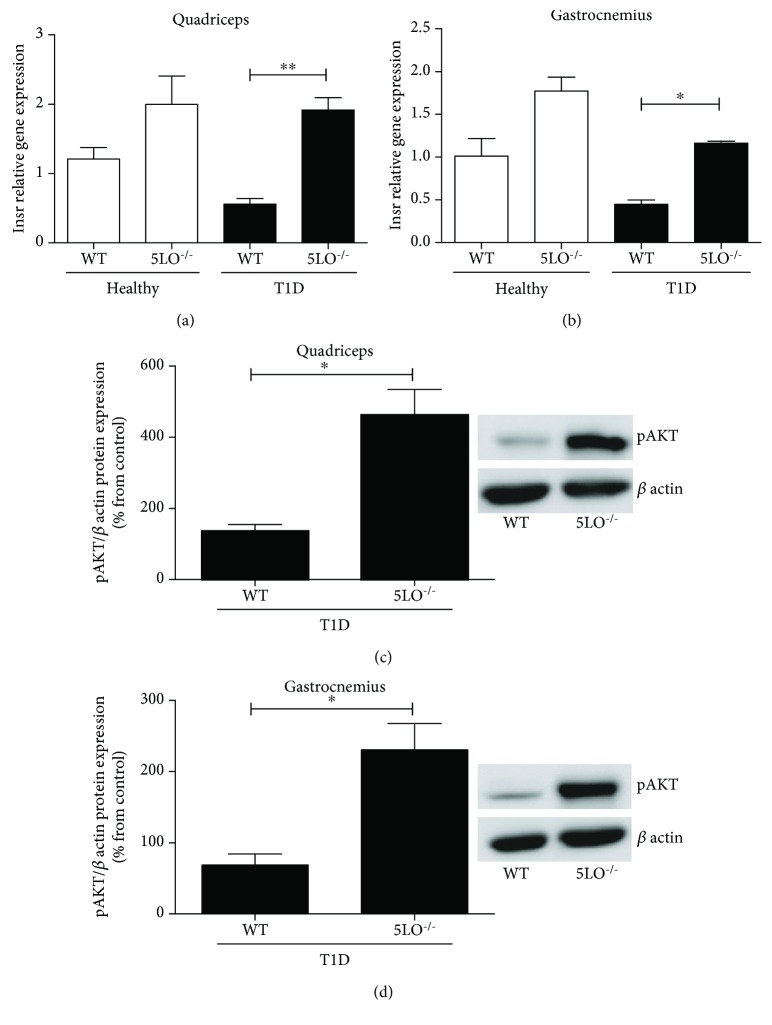
Insulin pathway in T1D muscle. The expression of the insulin receptor gene (*Insr*) in muscle homogenates of quadriceps (a) and gastrocnemius (b). Phosphorylation of AKT in quadriceps (c) and gastrocnemius (d). *n* = 4-6 animals in each group. For western blot, *n* = 3. Values are the mean ± SEM. ^∗^*p* < 0.05; ^∗∗^*p* < 0.01.

The gene expression of the insulin receptor in homogenates of the quadriceps and gastrocnemius muscles was not significantly different between WT and 5LO^−/−^ mice. However, in diabetic 5LO^−/−^ mice, the expression of the insulin receptor was significantly higher than in diabetic WT mice (Figures 3(a) and 3(b)). In Figures 3(c) and 3(d), we can see that after insulin administration to diabetic and healthy mice, the muscles from diabetic 5LO^−/−^ have higher levels of pAkt than WT diabetic mice.

### 3.3. Muscle-Associated Macrophage Profile in T1D

**Figure 4:**
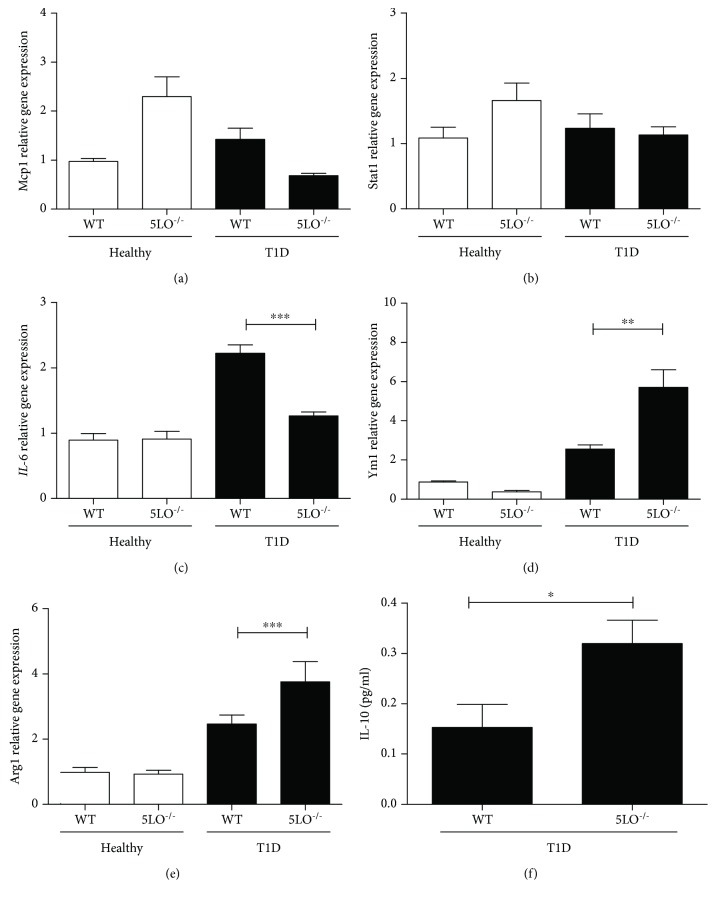
Muscle-associated macrophage profile in T1D. Quadriceps homogenates from diabetic mice were analyzed for the expression of anti-inflammatory markers (*Ym1*, *Arg*, and IL-10) and proinflammatory markers (*Mcp1*, *Stat1*, and *IL-6*). *n* = 4-5 animals in each group. Values are the mean ± SEM. ^∗∗^*p* < 0.01; ^∗∗∗^*p* < 0.001.

To assess inflammation in T1D mouse muscles, we analyzed the expression of some markers of the macrophage profile in quadriceps and gastrocnemius muscles. Homogenates of these tissues were obtained, and the mRNA expression of macrophage markers was measured in the precipitate and that of cytokines in the supernatant. We show in Figure 4 the results obtained in the muscle quadriceps. A similar pattern was observed in the gastrocnemius (data not shown).

The expression of proinflammatory macrophage markers *Stat1* and *Mcp-1* (CCL2) did not vary between the groups. However, the expression of the proinflammatory cytokine *IL-6* was significantly increased in the muscles from diabetic mice but only in those able to produce LTs (Figures 4(a)–4(c)). The expression of the anti-inflammatory macrophage markers *Ym1* and *Arg1* and of the anti-inflammatory IL-10 (Figures 4(d)–4(f)) was higher in diabetic 5LO^−/−^ mice compared to diabetic WT.

## 4. Discussion

The mechanisms involved in insulin resistance have been well described in T2D, but in T1D those mechanisms are less known. In the present study, we investigated the involvement of LTs in the development of insulin resistance in the muscle, a metabolically active tissue. It was found that in diabetic mice, LTs downregulated the insulin signaling pathway in muscles of mice pretreated with insulin. In addition, LTs shifted the muscle-associated macrophages towards the proinflammatory phenotype.

T1D was induced chemically using STZ, an antibiotic that kills the insulin-producing beta cells in the pancreas, thus inducing a deficiency in insulin production, insulin resistance, and chronic inflammation of the pancreatic islets. Because these symptoms resemble those of T1D in humans, the use of STZ has become popular as a model of T1D in rats and mice [21]. In 2015, Wu and Liang [22] showed that a single dose of STZ, although not enough to induce insulin resistance, was able to maintain hyperglycemia. We adapted the original model by giving five doses over 1 week, and after the last dose, all mice lost significant weight, exhibited polyuria, and had high blood glucose levels. In addition, we observed the phosphorylation of AKT in the muscle, which is indicative of insulin resistance.

Insulin resistance is a pathological characteristic of numerous metabolic diseases. To confirm that LTs produced during T1D promote insulin resistance, the response of diabetic WT and 5LO^−/−^ mice to insulin was initially evaluated. We observed that after a single dose of insulin, diabetic 5LO^−/−^ mice had a greater reduction in glycemia than WT diabetic mice. Li et al. [10] and Spite et al. [18] have shown that in T2D, the activation of the LTB4 receptor BLT1 leads to the downregulation of the insulin receptor cascade, blocking the action of the insulin receptor substrates and leading to insulin resistance. The same pattern was observed in our study. We observed that even with low levels of serum insulin, diabetic 5LO^−/−^ mice exhibited higher insulin receptor gene expression in both muscles tested. After treatment with one dose of insulin, only diabetic 5LO^−/−^ mice were able to recover the phosphorylation of AKT, an important molecule in the insulin signaling pathway. These data suggest that similarly to what happens in T2D, LTs also affect the insulin signaling cascade in T1D.

It has been described that in skeletal striated muscle tissue, including the quadriceps and gastrocnemius, T2D induces the expression of the proinflammatory cytokine IL-6 and a reduction in IL-10 levels [25]. Our results show that this also occurs in T1D; in addition, we showed that T1D mice unable to produce LTs (5LO^−/−^) produced a higher amount of the anti-inflammatory cytokine IL-10, which indicates that in diabetic mice, in the absence of LTs, muscle-associated macrophages acquire an anti-inflammatory profile. Kim et al. [26] showed that IL-6 decreased skeletal muscle insulin action and signaling, which corroborates our results showing that higher IL-6 expression was correlated with a lower expression of the insulin receptor and AKT phosphorylation in the muscles of diabetic WT mice. Hong et al. [25] showed, in a model of diet-induced insulin resistance, that treatment with IL-10 prevented insulin resistance and increased AKT phosphorylation. These findings are similar to those found in the muscles of the diabetic 5LO^−/−^ mice in the present study, where increased pAKT and IL-10 levels were observed. In addition, in a previous study it was shown that T2D mice have higher systemic levels of LTB4 than healthy mice [20]. Together, these results suggest that in diabetic mice, increased LT levels downregulate the insulin signaling pathway, thereby leading to insulin resistance in muscles. They also help us to understand the mechanisms responsible for the development of insulin resistance in T1D with the involvement of LT.

It has been shown recently that in peritoneal macrophages from T1D 5LO^−/−^ mice, the expression of anti-inflammatory macrophage markers (Arg1 and Ym1) was enhanced, whereas the WT macrophages expressed higher levels of proinflammatory markers. Also, systemic inflammation is less intense in T1D 5LO^−/−^ mice compared to the WT. This indicates an important role for LTs in the development of systemic inflammation in diabetes and reprogramming of tissue macrophages towards a proinflammatory phenotype [26]. Here, we show that macrophages resident in muscles are similarly reprogrammed by LTs in T1D mice.

## Data Availability

The values behind the means, standard deviations, and other measures reported in the data supporting the findings of this study can be obtained from the corresponding author upon reasonable request (Dr. Joilson de Oliveira Martins, martinsj@usp.br).
